# Drug testing on arrest–who benefits?

**DOI:** 10.1186/s40352-019-0103-z

**Published:** 2020-01-16

**Authors:** Marc Connor, Gill Green, Neale Thomas, Arun Sondhi, David Pevalin

**Affiliations:** 1 Office National Statistics, Segensworth Road, Titchfield, Hampshire, PO15 5RR UK; 2 University of Essex Colchester Campus, Wivenhoe Park, Colchester, Essex CO4 3SG UK; 3 Public Health & Wellbeing Essex County Council, County Hall, Chelmsford, CM1 1QH UK; 4 Therapeutic Solutions (Addictions), 4 Old Park Lane, London, W1K 1QW UK; 5 University of Essex Colchester Campus, Wivenhoe Park, Colchester, Essex CO4 3SG UK

**Keywords:** Strategic commissioning, Drug testing, Offender management, Drug treatment, Systems, UK

## Abstract

**Background:**

Drugs and crime are linked and diversion from the criminal justice system into drug treatment is a well-established policy response. The point of arrest is a pivotal moment to initiate a drug-specific intervention. This paper assesses the impact of the introduction of drug testing on arrest (DToA) into a low crime area in England. Our mixed methods study analysed performance data collected by the National Drug Treatment Monitoring/Drug Test Recorder datasets and feedback from a series of semi-structured interviews with both clients and professionals.

**Results:**

In total, 2210 people were tested 2861 times of which 42.0% (*n* = 928) tested positive. Of those, 3% subsequently engaged with effective treatment. However, throughout the criminal justice system, treatment engagements increased year on year from 20% (*n* = 77) to 26% (*n* = 131). Clients (*n* = 19) and professionals (*n* = 14) reported that DToA was an acceptable/tolerable addition to the treatment pathway. Interviews suggested that the point of arrest may help primary desistance from further offending.

**Conclusions:**

The staggered introduction of the DToA made direct measure of impact difficult and there appears to have been a ‘displacement’ effect in response to the extra investment. However, DToA appears to have contributed towards an overall uplift in criminal justice drug treatment system performance activities (identification, assessment, referral etc.) and may have served to help strengthen care pathways. Barriers were noted about engagement with DToA by entrenched opiate users, suggesting that the effectiveness of DToA was limited within that group. This study is the first to investigate the impact of the introduction of DToA into a low crime area.

## Introduction

Whilst the links between drugs and crime are complex (Bennett 2000; Best, Sidwell, Gossop, Harris, & Strang, 2001; Gossop, Marsden, Stewart, & Rolfe, 2000), there is substantive evidence describing the association between drug treatment and crime reduction (Gossop, Marsden, & Stewart, 1998; Gossop, Trakada, Stewart, & Witton, 2005; Jones et al., 2009). The diversion of substance misusing offenders into effective drug treatment (NTA, 2006) is a key element within the UK Governments’ anti-drugs strategy (Home Office, 2017) and supporting people to address their dependence is therefore critical to tackle the risk of reoffending.

The Drugs Act of 2005 sets out provisions for the use of DToA for specified Class A drugs. DToA is targeted towards adults (18 yrs.+) who have been charged or convicted of a ‘trigger offence’. These offences are primarily acquisitive and include all theft and drug related offences e.g. handling/taking without consent and possession/intent to supply (Home Office, 2010). In addition, an arrestee may be tested on the Inspector’s Authority if his or her offending behaviour is thought to be drug related. Implicit in the UK model is the use of quasi-compulsory components (the Required Assessment and Restrictions on Bail) that mandate engagement with community treatment services. The use of legislative levers has been questioned, with commentators arguing that there has been a shift from a public-health model towards a more punitive “criminalisation” or a “criminal justice turn” away from treating harm to drug users to managing harm (in terms of offending) committed by drug users (Hunt & Stevens, 2004; Seddon, Ralphs, & Williams, 2008; Seddon, Williams, & Ralphs, 2012; Stevens, 2007; Stimson, 2000). This ‘reframing’ of the interaction between substance misuse and the criminal justice system envisages a greater role for police as brokers for offenders to access and engage with treatment (Duke, 2006, 2013). The current UK anti-drug strategy remains supportive of the use of DToA to divert offenders into drug treatment as an anti-crime initiative (Home Office, 2017).

Relatively high levels of class A drugs misuse, primarily opiate (heroin) and/or crack-cocaine (OCU), have been observed throughout the criminal justice population, For example, the UK Boreham et al (2007)–06 reported that 26% of all respondents had taken heroin and/or crack within the month prior to their arrest (Boreham, Dollin, & Pudney, 2007) and a longitudinal study of newly sentenced prisoners (*n* = 1457) reported that up to a third of participants had taken OCU within the year prior to their incarceration (Stewart, 2009). Such observations have provided the impetus for the introduction of a range of policies and interventions in many parts of the world that aim to divert offending drug users into drug treatment to reduce drug-related harm. Evidence supporting the effectiveness of such interventions is mixed (McSweeney, Turnbull, & Hough, 2002). A systematic review and meta-analysis of the effectiveness of these types of diversionary programmes identified 16 studies (11 in North America, 4 in the UK and 1 in Australia) published between 1985 and 2012 (Hayhurst, Leitner, et al., 2015) and a further four studies (3 in the US and 1 in Australia) published between 2012 and 2016 (Hayhurst et al., 2017). The review found evidence of a small impact in terms of reducing drug use, but the evidence supporting a reduction in offending was uncertain (due to lack of outcome measure comparability). The studies were characterised by poor methodological quality linked to modest sample sizes, high attrition rates, retrospective data collection, limited follow-up, and no random allocation. There was also evidence of publication bias in favour of studies reporting statistically significant outcomes (Hayhurst et al., 2015). Generalisability of the findings to other settings, such as England, was also a major limitation as over 99% of participants included in the review were from California and the majority were methamphetamine users, a drug that accounts for only 0.1% of the English drug treatment population.

A systematic review and meta-analysis on the effects of European drug treatment programmes on reoffending found significant positive overall effects of treatment on both crime and drug use (Koehler, Humphreys, Akoensi, Sánchez de Ribera, & Lösel, 2014). These studies, from six countries but mostly from the UK, primarily evaluated substitution-based therapy. The international evidence thus suggests that diversion and treatment may help a minority to reduce drug use and offending behaviour, although the cost-effectiveness is unclear, and the quality of the evidence is not strong. Both reviews note the need for better quality large-scale evaluations. Specifically, they highlighted the need for studies to examine which groups of class A drug-using offenders were most likely to benefit from diversionary interventions (Hayhurst et al., 2015).

Treatment interventions are aimed at helping with desistance from further criminal activity. Concepts of desistance are placed on a continuum from ceasing offending and ‘staying stopped’ (Maruna, 2005) to a reduction of the level of offending albeit with potential for relapse (Burnett, 2004). Sampson and Laub (2005) describe a life-course theory to establish the importance of “turning points” that create prosocial changes that interrupt a criminal career. This model emphasizes childhood and the role of school, family, and an offender’s social environment. Farrell and Maruna (2004) describe *primary* (first changes in behaviour) and *secondary* (shifts in identity) levels of desistance which are supported by interventions aimed at addressing antisocial and criminal behaviours. McNeill (2006) argues that a “desistance paradigm” is grounded within the offender-therapist relationship to facilitate behaviour change. Therefore, introducing interventions at key ‘turning points’, such as the point of arrest, can be viewed as initiating the primary desistance process, potentially leading to further long-term changes in offender criminal identities.

### Drug testing in police custody in the UK

Studies have consistently highlighted the arrest event as a key point to initiate a drug-specific intervention (Hunter, McSweeney, & Turnbull, 2005; Oerton et al., 2003; Sondhi, O'Shea, & Williams, 2002). Consequently, mandatory DToA was introduced to divert detainees to treatment, ensure compliance with existing programmes and to develop an early warning system to prevent relapse (Harrell & Roman, 2001). The ‘Tough Choices’ policy was initiated by the Drug Interventions Programme, which itself had been introduced into the sixty six areas identified with high crime and high OCU prevalence during the early 2000s (Collins, Cuddy, & Martin, 2017; Mallender, Roberts, & Seddon, 2002; NTA, 2011; Skodbo et al., 2007). Tough Choices targets a wider range of detainees for referral to drug treatment. Any person arrested for a ‘trigger offence’ is automatically subject to a drug test for OCU. If the test is positive, the supervising custody officer makes an appointment with the community Criminal Justice Intervention Team (CJIT) for the client to attend a required assessment (RA). During the RA interview, the key worker gauges treatment need based upon the type and level of substance misuse. Failure to attend the RA is a breach of bail conditions and leads to further sanctions such as fines, restriction on bail or imprisonment. Descriptions of a DIP ‘model’ have been hard to define (Turnbull & Skinns, 2010) as CJITs have increasingly been incorporated within local Integrated Offender Management (IOM) models aimed at supervising offenders in the community across a range of statutory and non-statutory services (Home Office/Ministry of Justice, 2015).

### Evidence of impact

Despite the widespread use of DToA as an intervention, there have been few published studies about the effectiveness of this approach (Bennett et al., 2008; Holloway, Bennett, & Farrington, 2006; Singleton, 2008). An evaluation of mandatory drug testing pilots in Scotland suggested that whilst they successfully targeted vulnerable and at-risk groups, lower than expected numbers of eligible detainees were recruited to the programme and subsequently referred to treatment. This appears to be largely due to implementation issues such as availability of treatment places, disparate management information systems and inadequate resources targeted towards police administrative responsibilities, especially with respect to the time required to complete a DToA test and referral (Skellington, McCoard, & McCartney, 2009). More people were tested but with diminishing returns in terms of costs for every additional drug user identified and reductions in re-offending. The effect of more limited impact as a result of drawing more people with lower drug using or offending profiles into the system has also been noted elsewhere (McSweeney, 2015; Singleton, 2008; Skellington et al., 2009).

Two recent UK studies compared the health and crime outcomes of those identified via DToA or by conventional Arrest Referral (McSweeney, 2015) and those compliant or noncompliant with the RA process (McSweeney, Hughes, & Ritter, 2018). The first found that DToA was less effective in engaging people into effective treatment and the second reported that there was no association between compliance and treatment engagement. Both concluded that DToA was not associated with improved health or crime outcomes.

Since 2011, DToA has expanded throughout the UK into designated non-intensive or ‘low crime’ areas on a voluntary basis and, given the mixed evidence about the programme’s effectiveness, we identified a need to evaluate its impact in this type of setting, in this case a large county in the South of England. Interviewing arrestees is recommended when evaluating local programmes that aim to change drug use behaviours (Bennett, 1998). Our focus was towards DToA impact on system performance and its acceptability with practitioners and clients rather than on individual health and crime outcomes. The low crime area reported here is a high performing and cost-effective recovery partnership, comprised of a consortium of stakeholders drawn from criminal justice, health, local authority third sector agencies.

### Research questions

- *RQ1*. To what extent was the DTOA program successfully implemented?
- *RQ2*. Is point of arrest an acceptable time for mandatory testing from the perspective of those delivering and receiving the test?
- *RQ3*. Which types of clients are more or less likely to engage with treatment when tested at arrest?

## Methods

The DToA ran in pilot form at a single custody suite during 2014 and became operational throughout the Police Force Area (nine custody suites in total) on 1st April 2015. The pilot site was chosen because of a surge in shoplifting in that area and because it had recently been modernised to incorporate DToA. System performance data were sourced from the Police’s Drug Test Recording (DTR) database and the Community Criminal Justice Report (CCJR), respectively. Police staff organise the DTR, which monitors individuals from arrest to assessment with the CJIT team. The CJIT team organises post assessment data. Both datasets are transmitted to the National Drugs Evidence Centre (NDEC) for processing. The CCJR, published via the National Drug Treatment Monitoring System (NDTMS), reports activities on a monthly, quarterly and annual basis.

**Fig. 1:**
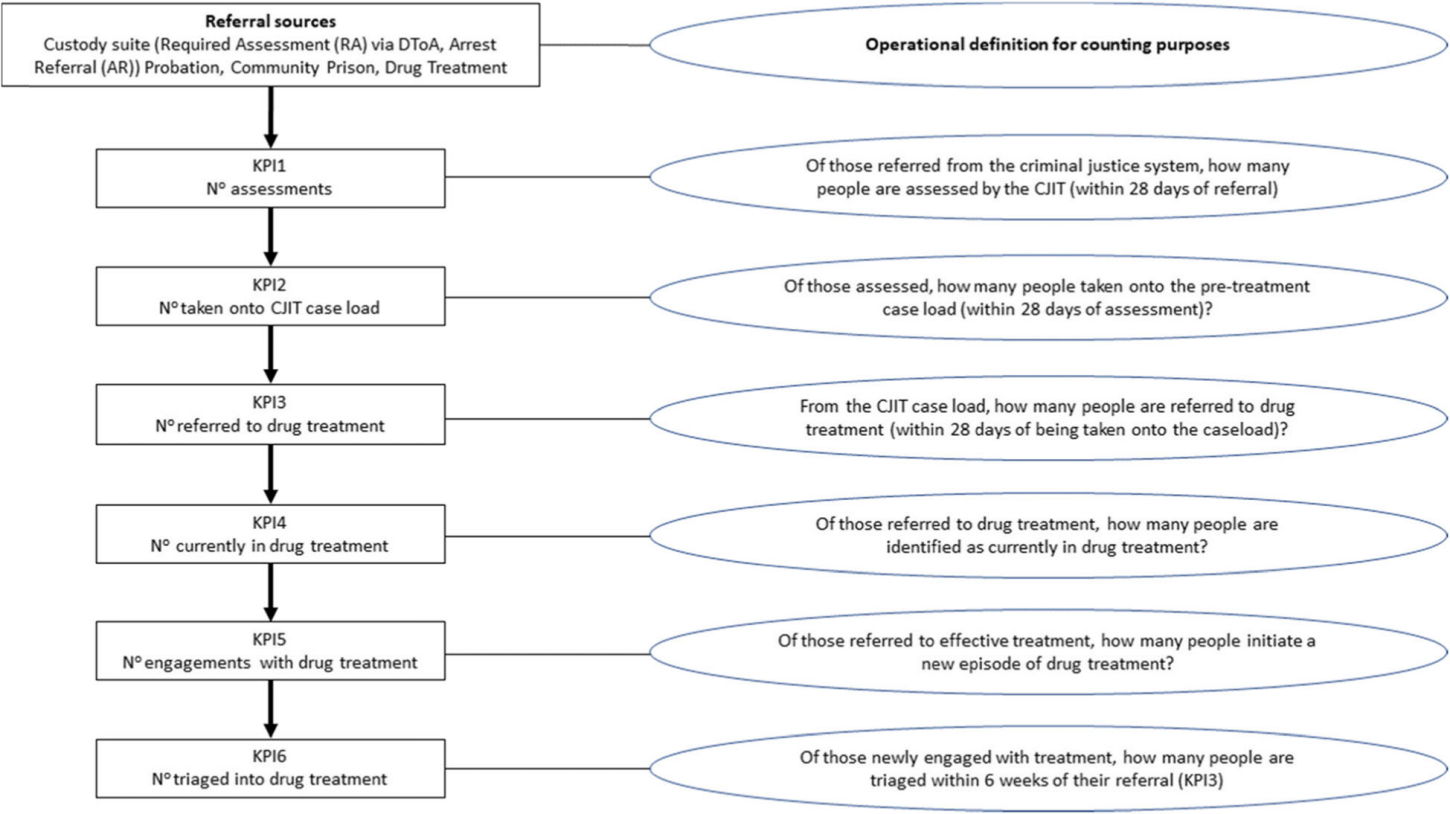
Key performance indicators (KPI), in sequential order, linked to their operational definitions as per the CCJR

For RQ1, we first compared the local demographic/drug misusing profile and positive drug testing cohort with the national averages (Home Office, 2015). To enumerate our assessment of impact, in both absolute and proportional terms, we compared the year on year change in six of the system key performance indicators (KPI) as prescribed and reported by the CCJR. Specifically, we focussed towards numbers: of assessments (KPI1); taken on the CJIT caseload (KPI2); of referrals to treatment (KPI3); in treatment (KPI4);, new to treatment (KPI5); and triaged within 6 weeks of referral (KPI6) (Fig. 1). Due to operational issues associated with delivering DToA throughout a large geographical area, and within a compressed time-frame, data were unavailable for quarter 42,014. The measures of impact we present here cover quarters 1, 2 and 3 for years 2014 and 2015, respectively.

To answer RQ2 & RQ3, data were collected via semi-structured interviews with 19 clients and 14 professionals. The perspectives of both groups were important because DToA needed to be acceptable to all involved if it were to be continued and we wished to identify any differential impact on different types of client. The sample of clients was comprised of 15 people who engaged into treatment via the DToA and 4 people enrolled on the Integrated Offender Management (IOM) scheme but who had tested negative. Candidates for interview were identified if they appeared on the DTR on more than one occasion. In total, 27 clients consented to interview but eight people did not attend their scheduled appointments. Interviews were conducted at the IOM offices by the research team and lasted for up to 1 hour. Client interviews covered the individual’s experience following arrest, specifically the acceptability of being tested at that time, their treatment and/or offending history. Interviews with professionals focused upon the acceptability of the scheme and their perception of the impact of DToA on clients. All participants signed informed consent sheets detailing the research aims and objectives. In addition, they were given a voucher to cover their expenses. Interviews were recorded, transcribed and a thematic analysis was conducted by two of the authors with qualitative expertise using an inductive and realist approach based on standard inductive thematic analysis (Braun & Clarke, 2006). A theme was defined as “a patterned response or meaning within the data set” (Braun & Clarke, 2006), p. 11. Codes were grouped together, and initial theme titles were generated. This was firstly done independently by the two co-authors to identify preliminary codes. These were then shared and discussed focussing on commonalities and differences between the codes of both authors to determine key themes. We also identified any clear differences in attitudes towards, and experiences of, DToA among the client group in terms of their age, service history and drug use. Authorisation to undertake the research was obtained from a University ethics committee (ref. 14016a) and the National Offender Management Service (NOMS) (ref.2015–234).

## Results

### Profiles of people tested, and clients interviewed

Between 1 April and 31 December 2015, 2210 individuals were tested 2861 times for opiates and/or crack-cocaine misuse. Of these, 928 (42%) tested positive compared to 35% reported in the national data (Home Office, 2015). Those tested were predominantly male (80.5%) and relatively young (18-22 yrs., 24%) but people who tested positive tended to be older (33-37 yrs., 20%). Whereas our demographic findings were comparable with those observed in the national data, our drug profiling observations were not. In our sample, 51% of people tested positive for crack-cocaine use only, 12% for opiate use only and 38% for both. The corresponding figures reported by the national data are 38%, 27% and 45% respectively (Home Office, 2015).

The average age of the 19 people interviewed was 37 years and 14 were male. Opiate use only was reported by 10 interviewees and in combination with crack-cocaine use in 2 cases. Most of those interviewed were either in or had experienced treatment (*n* = 15). The average age of those not attending their interview (*n* = 8) was 35 years of which 7 were male. Most of this group tested negative (*n* = 6).

*RQ1 -* To what extent was the DTOA program successfully implemented?

Analysis of the year on year change in the numbers and rates of people entering the criminal justice drug treatment pathway describes an expanding system, towards which the DToA significantly contributed (Table 1). The strategic stakeholders who were interviewed felt that the improved system performance was the result of the good level of police engagement with the DToA project, the strength of the partnership arrangements and the leadership demonstrated in the implementation of DToA.

**Table 1:** System performance activities associated with opiate and/or crack/cocaine users (OCU) in contact with drug treatment via the criminal justice system referral pathways

Key performance indicators (KPI)	2014	2015	Test*
	DToA (All) %	DToA (All) %	χ^2^ (DToA vs AOR)
(1) Assessments	40 (379) 11%	239 (513) 47%	131.7**
(2) Taken onto caseload	39 (378) 10%	158 (418) 38%	80.5**
(3) Referred to treatment	11 (152) 7%	46 (241) 19%	30.1**
(4) In treatment	2 (49) 4%	13 (69) 19%	5.6*
(5) New to treatment	9 (101) 9%	33 (172) 19%	5.2*
(6) Triaged within 6 weeks	5 (77) 6%	27 (131) 21%	7.4**

*p*-value * < 0.05 & ** < 0.01.

Notes: All – DToA = AOR and year = April 1st to December 31st

The total number of people assessed (KPI1), taken onto CJIT case load (KPI2) and referred to drug treatment (KPI3), increased from 379 to 513, from 278 to 418 and from 152 to 241, from years 2014 to 2015. Within those totals, the numbers of people identified by DToA increased from 40 (11%) to 239 (47%, *p* < 0.01), from 39 (10%) to 158 (38%, *p* < 0.01) and from 11 (7%) to 46 (19%, *p* < 0.01). The total number of people entering effective drug treatment (triaged within 6 weeks, KPI6) increased from 77 in 2014 to 131 in 2015. The DToA initiated 5 (6%) and 27 (21%) of these new effective treatment journeys in years 2014 and 2015 respectively (*p* < 0.01).

### RQ2 - Is the point of arrest an acceptable time for mandatory testing from the perspective of those delivering and receiving the test?

Many interviewees described the drug test as ‘*just another’* functional police activity. Participants were aware of the legislative sanction if they refused but there was general acceptance, or at least resignation, about the mandatory element of the RA: ‘*when you’ve tested positive, they say ‘do you agree’ and I’ve obviously said ‘yes’ and signed it and then an appointment is made. Last time … I was in there [police station] … .the sergeant was quite polite and asked me about the appointment time, not that it matters because I haven’t got a choice anyway’* (ID12).

There was uncertainty as to whether the referral to treatment was punitive or supportive. One participant said: ‘*I didn’t realise I suppose, that it was actually about supporting me if I had any problems’* (ID5). Despite reservations, many reported that it was a means ‘*to get help’* and were able to point to positive outcomes resulting from the mandatory requirement to attend treatment: ‘*I think it must have been first, second or third session I said ‘yeah, you know what, this is a blessing in disguise’. If I’d not got this treatment at the time I did, I probably would have ended back in jail. …*. *So yeah, I realised then that I needed help’* (ID8).

In general, there was concordance between the views expressed by professionals and clients. Clients’ mixed feelings about the mandatory nature of the test was reaffirmed by the police who reported a level of mistrust from the clients as ‘*it is forced upon them’* (ID52) and undertaken by the police. Two of the police officers interviewed described it as a ‘*box-ticking*’ exercise. However, they also reported that it was a helpful referral structure. According to a probation manager, ‘*We know that treatment works, and if this is a way that can get people faster and more people into that, then I think it would be beneficial’* (ID53).

Community practitioners reported that DToA resulted in more drug users receiving treatment including some who otherwise would not have sought it: *‘I think it gets them thinking and it does get them into treatment because whereas before if they didn’t get arrested and they didn’t test positive the chances are they probably would have never come to us’* (ID55). They described a number of clients whose test had resulted in very positive outcomes: *‘I had a DToA client who’s now on a DRR [Drug Rehabilitation Requirement] testing negative, engaging really well, really embraced all the support services around him … so it does work, I think it’s good’ (*ID56*).*

One of the police participants (ID58) criticised the way DToA identified people already known to the services on multiple occasions. He said, ‘ *… we’ll go through the motions. It’s going to be positive. We make them an appointment and they’re back in three weeks later and we’re going through exactly the same again. …*. *You just think well what’s the point?’* The community workers agreed that many of the clients were already known but noted that it was a useful process to alert both the client and their drug worker that current treatment was not working.

Overwhelmingly, clients reported a positive experience of being in treatment following referral. Rapport with the drug worker was paramount and they were described as: ‘*non-judgemental’* (ID1,7,8), ‘*relaxed*’ (ID1,11); ‘*friendly*’ (ID3,5,11), ‘*down to earth*’ (ID3,7); ‘*helpful*’; ‘*going the extra mile*’, ‘*really care’* (ID8); ‘*honest*’, ‘*easy to talk to*’ (ID12); ‘*make more time for you*’ (ID13); ‘*believes in me*’ (ID17). Continuity of contact was perceived as key to forge a trusting therapeutic relationship: ‘*I think the same key worker all the time is a big thing. I know that I have a good professional relationship with [drug worker] but it’s taken nearly 10 months for me to be able to talk to her because the truth hurts’* (ID16).

Despite some ambivalence, DToA was acceptable to, and often welcomed by, practitioners and acceptable or at least tolerable to clients. The range of views that were expressed ranging from *‘it’s essentially a box ticking exercise’* to being *‘a blessing in disguise’*, relates to the third aim about the differential benefits of DToA.

### RQ3 - Can one identify specific types that are more are less amenable to engage with treatment when tested at arrest?

There were differences between heroin users with a long history of use and treatment and cocaine users who generally did not have a long treatment history. For the former, offending and arrest were viewed as ‘normal’ and the drug test was just another ‘function’. They were more likely to question the need for a test if they were already in treatment. In contrast, some of those who tested positive for cocaine were new to the system and the community workers reported that DToA was particularly effective for clients who ‘*dabble*’. ‘*It’s because we’re picking up the cocaine users which normally wouldn’t come and ask for the support’ (*D60). And, as one of the cocaine users commented: ‘*But it is really when someone says this is a problem for you that you start to think about it’ (ID5).*

For many (mainly cocaine users), treatment was a learning experience including understanding the health effects of use. However, there was little evidence in the interviews that offenders used treatment to create a new lifestyle. Offending and drug use were described as part of ‘normal’ life and although DToA made a clear link between drugs and offending, participants who were longstanding users highlighted extraneous factors as key to changing lifestyle. This included prompts such as overdose or onset of health problems, as well as factors linked to stage in the life cycle. Ageing might prompt a reassessment of lifestyle and act as a motivation to engage with treatment*.*

Whilst some of those already receiving drug treatment reported that DToA was useful as a ‘wake up’ call and that they needed to engage more proactively with treatment, there were others who felt that the test had no benefit whatsoever. One client, who had had multiple tests described it as: *‘An absolutely pointless task’* (ID16). People with an earlier treatment history had mixed views. Re-engagement with treatment was welcomed by some but resented by others.

People naïve to treatment were the most positive about DToA, with one participant (ID1) saying, ‘*I was pretty happy to be honest, I can’t really fault it’*. One even described it as transformative: *‘[I was in] a dark, dark place. I’ll be honest with you, I’ve started feeling stuff, smelling stuff, hearing things, all the colours have all come back, it’s profound the difference in such a short period of time* (ID2).

## Discussion

### Main findings and implications

Our findings show that DToA contributed markedly towards a system wide increase in the identification, assessment and referral (treatment initiation activities) to drug treatment within a criminally active OCU cohort. And the introduction of DToA positively influenced the rate of OCU engagement with effective treatment but the direct impact was less than we expected. The implementation and process of running DToA was smooth and rapid. Strong leadership, support from senior management and working in partnership were integral to successful delivery. Both clients and providers felt that DToA produced speedier referrals. Thus, from a policy perspective, we propose that the introduction of DToA into this low crime area had a positive impact because it contributed towards effectively identifying opiate and crack-cocaine users, including those not currently or never in treatment, and referred them quickly for assessment and treatment.

The delivery of DToA was broadly acceptable to professionals and clients. The scheme was well regarded by most professionals although some reservations were expressed, and modifications suggested. DToA was also viewed as acceptable (or at least tolerable) to most clients providing its purpose was explained. DToA appears to have a differential impact upon those new to treatment and cocaine users compared to longstanding opiate service users. The former were more positive about engaging with services whereas the latter were more likely to be resigned to the process and attend because they ‘have to’. The limited impact on this group would appear to support McSweeney et al. (2018) finding about a lack of difference in treatment engagement between those who completed the required assessment and those who did not. Nevertheless, the mandatory nature of some referrals did have initial benefits by ensuring initial engagement with treatment and at times this facilitated strong worker-client relationships. Ensuring operational systems are aligned to create a therapeutic alliance is an integral component in facilitating engagement with treatment and other ancillary services (Joe, Simpson, Dansereau, & Rowan-Szal, 2001; Meier, Barrowclough, & Donmall, 2005). Moreover, the start of an effective therapeutic relationship begins the process of ‘primary’ desistance encouraging initial changes in behavior as described by Maruna and Farrall (2004).

The benefits for those already in treatment were unclear and some clients and professionals alike expressed reservations. Whilst both groups reported that a positive test provided a cognitive inflection point about the link between drugs and crime and the negative consequences of drug use, in isolation, it did not appear to be transformative leading to secondary desistance changes (Maruna & Farrall, 2004). Rather, it was accepted as normality. Clients identified external events (such as key relationship changes or overdose) as triggers to reconsider lifestyle changes. This finding may confirm the role of social bonds as a means to encourage desistance (Sampson & Laub, 2005) and suggests that interventions at the point of arrest might consider highlighting the consequences of drug use on familial relationships to affect behaviour change.

The differential impact, according to whether one takes a systems/policy or a client centred perspective, explains why the results of this study contradict the findings reported elsewhere such as the lower impact of DToA compared to conventional ‘cell-sweep’ referral (McSweeney, 2015) and comparing compliance with the required assessment following DToA (McSweeney et al., 2018). Crucially, these studies focussed on long-term treatment, health, and offending outcomes, while our research focused on the impact of DToA in terms of system performance at the point of initial contact and the period leading up to engagement with effective (NDTMS) treatment journeys. We did not investigate whether identification and referral via DToA resulted in more effective treatment outcomes than those referred via other routes, although the limited impact reported by many of the clients suggests it may not have.

Broadly, our client profile matched the patterns reported in the high crime areas but with one notable exception. In our sample, opiate use only was markedly lower, and crack-cocaine use only was markedly higher. Crack-cocaine only users are not suited to or warrant effective (NDTMS) treatment (Morgan, Heap, Elliott, & Millar, 2016) which suggests that, in low crime areas, the pool of people available for treatment, as recorded by NDTMS, may be limited.

From a systems perspective, our primary concern was with the low conversion rates of positive tests to effective treatment engagement. From the national outputs, we estimated that in the region of 20% of people testing positive engaged with effective treatment (Home Office, 2015; National Archies, 2019) but in our sample, only 3% of those testing positive did so. Our observed low conversion rates may be symptomatic of the atypical (compared to high crime areas) drug profile of our cohort and suggests that, for DToA to be successful in low crime-high levels of crack-cocaine misuse, the treatment offer might require some adjustment or that DToA in general become more responsive within its operational context.

Other contributory factors towards low conversion rates may include a system at or near capacity and limited performance oversight.

### Limitations of the evaluation

Quantifying the direct impact of the introduction of DToA was technically confounded by the staggered delivery of the intervention. Data reporting during the training and delivery phase (quarter 4, 2014) was sporadic, leading to the decision not to include that and the corresponding period of 2015 within this report. This highly likely reduced the sample size available in both years, and almost certainly did so with respect to 2015. Interviewees were conveniently sampled, and it is likely that those who agreed to be interviewed were those who had had a more positive experience and engaged better with treatment. Future studies might opt for a longer time-frame (post introduction) within which to evaluate impact. This would allow for a time series approach re the quantitative investigations and the qualitative arm to sample at defined points e.g. pre, during, one and two-year post the introduction of DToA. We did not have full access to the two-year data, and it would be useful to understand to what extent (1) conversion to treatment rates are influenced by performance frameworks and/or adjusted treatment offers and (2) as the intervention matures, do people’s views and perceptions reflect the inherent stability brought about by longevity. Conducting the interviews within IOM premises may have influenced the client narratives.

## Conclusion

The introduction of DToA into our low crime area had an impact and not in the way we initially expected. In the high crime areas where mandatory DToA was introduced, it superseded the Arrest Referral (‘cell sweeping’) programmes, which had operated on a voluntary recruitment strategy involving the key worker asking the client if they would like to engage with the drug recovery services. In our study, DToA appeared to develop as an ‘add on’ or complementary service to the existing Arrest Referral activities, thereby facilitating a system wide strengthening of the criminal justice referral pathways into drug treatment.

The delivery of DToA was broadly acceptable to professionals and at least tolerable to clients and there was positive feedback stressing the importance of the therapeutic alliance. The study highlighted opportunities to develop primary desistance models at the point of arrest that encourage offenders to examine prosocial bonds associated with illicit drug misuse (such as maturation and the wider effect on families). Such a model would need to consider how to address negative attitudes from entrenched opiate users who may not be at a ‘turning point’ to consider behavioural changes.

Should other low crime areas be thinking about introducing DToA, we suggest several modifications. We recommend a targeting model from the outset so that resources are rationalised and client/professional frustrations of ‘over testing’ of people already known to them are minimised. In addition, redeploying drug workers to undertake the drug test would enhance system efficiencies and, perhaps more importantly, strengthen the drug worker-client relationship at an earlier stage, thereby maximising the opportunities for future engagement with effective treatment.

Whilst identification of drug use and rapid referral to treatment is not a panacea for substance misuse or offending, it is nevertheless an important step for those who are treatment naïve, and our findings are likely to be transferable to other ‘low crime’ areas. That said, the benefits from the client perspective are ambiguous, particularly for longstanding drug-using offenders. DToA currently offers them little and we would suggest the development of a specific pathway for longstanding drug users already in treatment to ensure that they engage with their key worker at the earliest possible stage in the process.

## Data Availability

The datasets used and/or analysed during the current study are available from the corresponding author on reasonable request.

## Abbreviations

AOR: All other Referrals
AR: Arrest Referral
CCJR: Community Criminal Justice Report
CJIT: Criminal Justice Intervention Team
DIP: Drug Interventions Programme
DToA: Drug Test on Arrest
DTR: Drug Test Recorder
HO: Home Office
KPI: Key Performance Indicator
NDEC: National Drugs Evidence Centre
NDTMS: National Drug Treatment Monitoring System
NTA: National Treatment Agency for Substance Misuse
OCU: Opiate and/or Crack-cocaine User
PFA: Police Force Area
PHE: Public Health England
RA: Required Assessment
RoB: Restrictions on Bail
